# Should Physicians Be Aware of Rhythm Disturbances in Adults with Systemic Autoimmune Diseases and Anti-Ro52 Antibodies? A Cross-Sectional Study

**DOI:** 10.3390/jcm13123510

**Published:** 2024-06-15

**Authors:** Javier Gamazo-Herrero, Julio Antonio Medina-Luezas, Ivan Cusacovich, Miguel Martín-Asenjo, Carmen González-Montagut-Gómez, María Dolores Sánchez-González, Francisco Aramburu-Muñoz, Iustina Janta, Emilio García-Morán, Carlos Miguel Veras-Burgos, Luis Corral-Gudino, Cristina Abad-Molina, Roberto González-Fuentes

**Affiliations:** 1 Systemic Autoimmune Diseases Unit, Department of Internal Medicine, Hospital Clínico Universitario de Valladolid, Gerencia Regional de Salud de Castilla y León (SACYL), Avda. Ramón y Cajal nº3, 47003 Valladolid, Spain; jgamazoh@saludcastillayleon.es (J.G.-H.);; 2 Systemic Autoimmune Diseases Unit, Department of Rheumatology, Hospital Clínico Universitario de Valladolid, Gerencia Regional de Salud de Castilla y León (SACYL), Avda. Ramón y Cajal nº3, 47003 Valladolid, Spain; 3 Department of Medicine, Dermatology and Toxicology, Medicine College, University of Valladolid, Avda. Ramón y Cajal 7, 47003 Valladolid, Spain; 4 Electrophysiology Unit, Department of Cardiology, Hospital Clínico Universitario de Valladolid, Gerencia Regional de Salud de Castilla y León (SACYL), Avda. Ramón y Cajal nº3, 47003 Valladolid, Spain; 5 Department of Internal Medicine, Hospital Universitario Río Hortega, Gerencia Regional de Salud de Castilla y Leon (SACYL), C/Dulzaina n°2, 47012 Valladolid, Spain; 6 Immunology Laboratory, Department of Microbiology, Hospital Clínico Universitario de Valladolid, Gerencia Regional de Salud de Castilla y Leon (SACYL), Avda. Ramón y Cajal nº3, 47003 Valladolid, Spain

**Keywords:** autoimmune diseases, electrocardiography, anti-Ro52 autoantibodies, adults, QT interval

## Abstract

**Objectives:** The association between anti-Ro/SSA antibodies and the appearance of cardiac rhythm disorders in adults is discussed. We aim to study this relationship, together with active treatments and comorbidities, and its impact on daily clinical practice in adults with systemic autoimmune diseases (SADs). **Methods:** This cross-sectional single-center study was conducted in a tertiary hospital between January 2021 and March 2022. A sample of adult patients followed up in the SAD Unit with a diagnosis of a SAD and previously tested for anti-Ro/SSA and anti-La/SSB were recruited. All of them underwent a 12-lead electrocardiogram. **Results:** 167 patients were included. 90 (53.9%) were positive for anti-Ro60, 101 (60.5%) for anti-Ro52, and 45 (26.9%) for anti-La/SSB; 52 (31.3%) were triple-negative. 84% were women, and the mean age was 59 years (standard deviation 12.8). The most common SAD was primary Sjögren’s syndrome (34.8%), followed by systemic lupus erythematosus (24.6%) and rheumatoid arthritis (22.8%). A statistically significant relationship was found between anti-Ro52 positivity and cardiac rhythm disorders (relative risk = 2.007 [1.197–3.366]), specifically QTc prolongation (relative risk = 4.248 [1.553–11.615]). Multivariate regressions showed a significant association, with diabetes mellitus being the most related comorbidity. The association between anti-Ro52 antibodies and atrioventricular conduction disorders was not significant. **Conclusions:** The presence of anti-Ro52 antibodies in adult patients with SADs is associated with an increased risk of QTc prolongation. Electrocardiographic screening of patients with SAD, anti-Ro52 antibodies, and other risk factors, like diabetes mellitus or QT-prolonging drugs, seems advisable. Those with baseline electrocardiogram abnormalities or additional risk factors should undergo electrocardiographic monitoring.

## 1. Introduction

Anti-Ro/SSA and anti-La/SSB antibodies are autoantibodies directed against 52, 60, and 48 kilodaltons (kDa) ribonucleoproteins. The coexistence of anti-Ro52 and anti-Ro60 antibodies and anti-Ro/SSA and anti-La/SSB antibodies is frequent in the same patient. Anti-Ro/SSA antibodies are clinically associated with various organ-specific and systemic autoimmune diseases (SADs), although they have also been detected in up to 2.7–8% of the general population [1,2,3].

The role of anti-Ro/SSA antibodies in pregnant women, with or without defined SADs, in the risk of neonatal lupus or congenital heart block by transplacental transfer is firmly established [4,5,6,7,8]. Two pathogenic mechanisms, which may overlap, have been described: first, dysfunction of L-type and T-type Ca^2+^ channels in fetal conduction tissue cells by interaction with these antibodies [9,10,11] and second, inflammatory-fibrotic tissue damage mediated by complement and autoreactive lymphocytes on apoptotic cardiomyocytes with externalized Ro and La nuclear antigens on their surface [12,13,14,15]. While the former mechanism is more associated with low-grade atrioventricular block (AVB) that is reversible with treatment, the latter better explains the worse prognostic forms of advanced AVB.

It had previously been accepted that adult cardiac conduction tissue was resistant to damage induced by anti-Ro/SSA and anti-La/SSB antibodies due to a larger pool of L-type calcium channels and a lower susceptibility to apoptosis than fetal cardiocytes [16] and, therefore, to the development of irreversible tissue damage. In contrast, numerous clinical cases and small case series have described atrioventricular (AV) conduction disturbances in adults with SADs and anti-Ro/SSA or anti-La/SSB antibodies, some with response to corticosteroids and immunosuppression, and recent studies have estimated a twofold increased risk of AV conduction disorders in the presence of anti-Ro antibodies, which forces questioning the previous postulate [17,18,19,20,21] (Supplementary Material Table S1). Considering the possible role of cell apoptosis and the externalization of Ro and La antigens to the surface of cardiomyocytes in neonatal lupus, it could be hypothesized that in adults carrying these autoantibodies, acquired myocardial damage of any origin (viral, ischemic, autoimmune) could facilitate their pathogenic action.

Anti-Ro/SSA and anti-La/SSB antibodies have also been associated with prolongation (more extended than 440 ms) of the QT interval corrected for heart rate (QTc) in adults [22,23]. Prolonged QTc interval is a risk factor for the development of ventricular arrhythmias, such as Torsade de pointes, and sudden death, especially if the QTc interval is longer than 500 ms [3]. Its development is the result of inhibition of voltage-dependent HERG potassium channels (Kv11.1), crucial in myocardial repolarization, due to the structural homology between its alpha subunit, especially in the pore area, and the Ro52 antigen [22]. Recent investigations have additionally demonstrated that anti-Ro52 antibodies reduce hERG channel expression [24]. Dysfunction of these channels is also implicated in congenital and pharmacological long QT syndromes. The frequency of prolonged QTc in patients carrying anti-Ro/SSA, specifically anti-Ro52, has been estimated to be between 3 and 10%, depending on the cutoff point chosen for the definition of prolonged QTc, significantly higher than in non-carrier patients [3,25]. In contrast, other authors have not observed this association [26,27]. Differences in the target population (e.g., patients with a specific SAD, antibody carriers with or without associated SAD) in the definition of QTc prolongation, in the sample size, in the distinction between specific anti-Ro/SSA antibody subtypes or in antibody titer strength, in addition to the consideration of comorbidities or medications, could explain these discordances (Supplementary Material Table S2).

Our study aimed to establish the prevalence of cardiac rhythm disturbances in patients with SADs and anti-Ro/SSA antibody positivity, determine their impact, and establish their implications for daily clinical practice. We also tried to evaluate the interaction of antibodies, comorbidities, and drug intake.

## 2. Materials and Methods

### 2.1. Type of Study

A single-center, observational, cross-sectional study was conducted between January 2021 and March 2022.

### 2.2. Patients

Adults between 14 and 80 years old fulfilling classification criteria for diagnosing a SAD under regular follow-up in the SAD Unit at Hospital Clínico Universitario in Valladolid, Spain, were the target population.

We calculated that 244 patients should be included to have an 80% power to show a relationship between the presence of anti-Ro/SSA or anti-La/SSB and cardiac rhythm disturbances at a 2-sided α level of 5% as the prevalence of prolonged QTc had been previously estimated by 12% in anti-Ro/SSA-positive patients and 3% in anti-Ro/SSA-negative patients [25].

Patients with seropositivity for anti-Ro/SSA (both anti-Ro60 and anti-Ro52) or anti-La/SSB antibodies were recruited by telephone from a list provided by the immunology laboratory in order not to lose sample size. Seronegative patients, more frequent in the clinic, were randomly included (the first seronegative patient seen daily by each physician during the recruitment period). Seronegative patient recruitment was slower than seropositive, so it could not be completed by the end of this period.

Regardless of their serological status, patients without a defined SAD were excluded. Patients with weak and intermittent positivity for anti-Ro/SSA or anti-La/SSB, as well as patients with spondyloarthropathy associated with HLA-B27, due to their predisposition to the development of atrioventricular conduction disorders [28], were also excluded.

### 2.3. Variables

Numerous epidemiological variables, family history, comorbidities, type of SAD, active pharmacological treatments, and immunological profile were collected after reviewing the patient’s clinical chart, referenced to the day of giving their informed consent and enrollment in the study (Supplementary Material Table S3). The “time since diagnosis of SAD” or “since first seropositivity” was considered to be the time that had elapsed from the first reference recorded in the clinical chart up to that moment. Due to the variety of SADs evaluated and the difficulty of using unique shared clinical activity or accrual damage indexes, these variables were not considered.

Cardiac conduction and rhythm were assessed employing a 12-lead surface electrocardiogram (EKG), performed on the day the informed consent was signed and blindly evaluated by experts from the Electrophysiology Unit of the cardiology service of our center. Heart rate, RR, PQ, QRS, and QT intervals were measured manually (absolute error of ±2 ms). The QTc interval was calculated automatically using the Bazett formula (QTc = QT/√RR). The electrocardiographic disturbances were classified into sinoatrial conduction disorders, AV conduction disorders (1st, 2nd, and 3rd-degree AVB), intraventricular conduction disorders, QTc prolongation, and tachyarrhythmias according to internationally recognized criteria [29,30,31,32]. We considered the QT interval to be prolonged if the QTc was longer than 440 ms [33], in order to make our results more comparable to previous investigations.

An immunoblot technique determined the immunological profile (EUROLINE ANA Profile 3 (IgG) Kit from Euroimmun) (Order No. DL 1590-5001-3 G) with automatic interpretation using the EUROBlotOne device (Euroimmun, Lübeck, Germany) and EUROLineScan software (version 3.2), obtaining a semi-quantitative result (negative, doubtful, weak positive, positive, and strong positive). For the analysis of the role of titer strength, we transformed these results into a dichotomous variable: strong positives and all other patients.

### 2.4. Statistical Analysis

Dichotomous qualitative variables were expressed as absolute and relative frequencies. After performing the normality Kolmogorov–Smirnov test, continuous quantitative variables were represented as mean and standard deviation or as median and interquartile range according to their distribution. We used the chi-square test (χ^2^) or Fisher test (if necessary) to assess the statistical association between categorical variables, while between categorical and continuous variables, the independent samples T-student test or the Mann–Whitney U test was performed.

First, a bivariate analysis by binary logistic regression was conducted between the variable “cardiac rhythm disorders” (any abnormality) and other variables of interest collected from the clinical chart. Those that were significant and had biological plausibility were included in a multivariate logistic regression model (“enter” method). This process was repeated with AV conduction disorders and QT prolongation. Statistical significance was set when the *p*-value was lower than 0.05 (95% confidence interval (CI)). All statistical analyses were performed with IBM SPSS Statistics software (version 25).

## 3. Results

### 3.1. Study Population

In the study, 167 patients were included; 83.8% were women, and most of them were Caucasian. The average age was 59 years (12.8). 58 patients (34.7%) had primary Sjögren’s syndrome (pSS), 41 (24.6%) systemic lupus erythematosus (SLE) and cutaneous lupus, 38 (22.8%) rheumatoid arthritis (RA), 10 (6.0%) scleroderma, and 39 (23.4%) other SADs. The most frequent comorbidity was hypertension (HTN) (31.7%), followed by chronic respiratory disease (19.8%), diabetes mellitus (DM) (8.4%), and chronic kidney disease (CKD) (7.2%).

**Figure 1:**
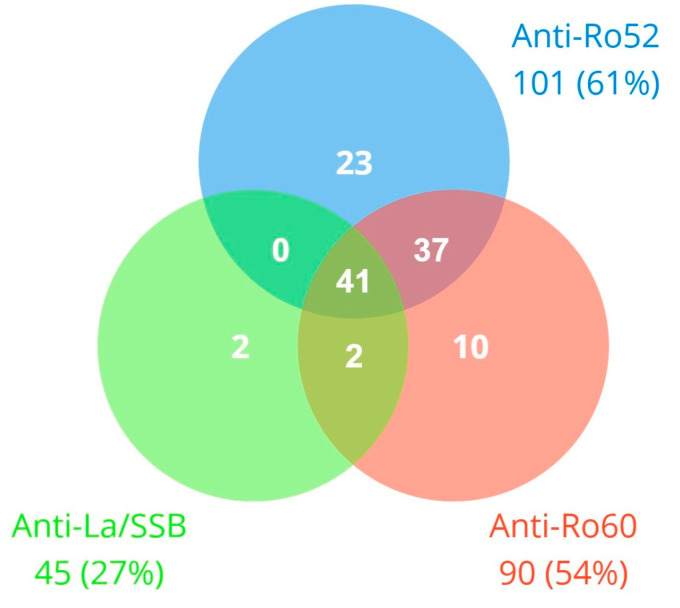
Simultaneous positivity for anti-Ro60, anti-Ro52, and anti-La/SSB antibodies (*n* = 115 patients with any positive).

We found 115 patients (68.8%) were positive for any of the autoantibodies under study: 90 (53.9%) were positive for anti-Ro60, 101 (60.5%) for anti-Ro52, and 45 (26.9%) for anti-La/SSB (Figure 1). Additionally, 52 patients (31.3%) were negative for any of these antibodies. Among the positives, 62 (37.1%) and 72 (43.1%) were strongly positive for anti-Ro60 and anti-Ro52, respectively (Supplementary Material Table S4).

**Figure 2:**
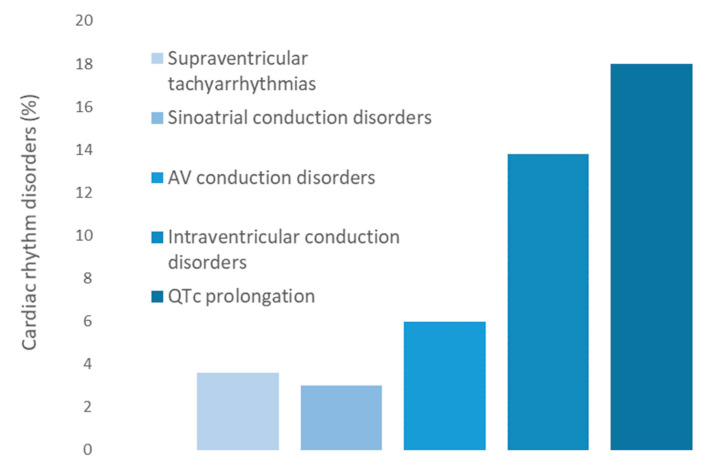
Cardiac rhythm disorders in the study (*n* = 57).

We found that 57 patients (34.1%) presented some abnormality in the surface EKG: 6 (3.6%) with supraventricular tachyarrhythmias, 5 (3.0%) with sinoatrial conduction disorders, 10 (6.0%) with AV conduction disorders (9 with a first degree AVB and 1 with a second degree AVB), 23 (13.8%) with intraventricular conduction disorders, and 30 (17.9%) with QTc interval prolongation (mean QTc = 454 ms, 95% CI (447, 461)) and 1 with U wave (Figure 2). All patients except one presented QTc values < 500 ms. He was a 70-year-old man with a diagnosis of SLE 27 years before with several risk factors for rhythm disorders such as active smoker state, hypertension, diabetes, CKD, antimalarial and β-blocker intake, and anti-Ro52 moderate-positivity. This patient had a QTc interval of 520 ms, in addition to a sinoatrial conduction disorder and a second-degree Möbitz I AVB. No patients developed ventricular tachyarrhythmias.

As mentioned above, anti-Ro52 antibodies have been the most frequently associated with AV conduction disorders and QTc prolongation in the literature. So, we grouped our patients according to anti-Ro52 status. Characteristics of the overall series and each group are shown in Table 1.

Anti-Ro52 carriers presented more frequently CKD (*p* = 0.022) or pSS (*p* < 0.001), while they had less frequently dyslipidemia (*p* = 0.021), active exposure to corticosteroids (*p* = 0.012) and immunosuppressants (*p* < 0.001), or a diagnosis of RA (*p* < 0.001).

### 3.2. Cardiac Rhythm Disorders

**Figure 3:**
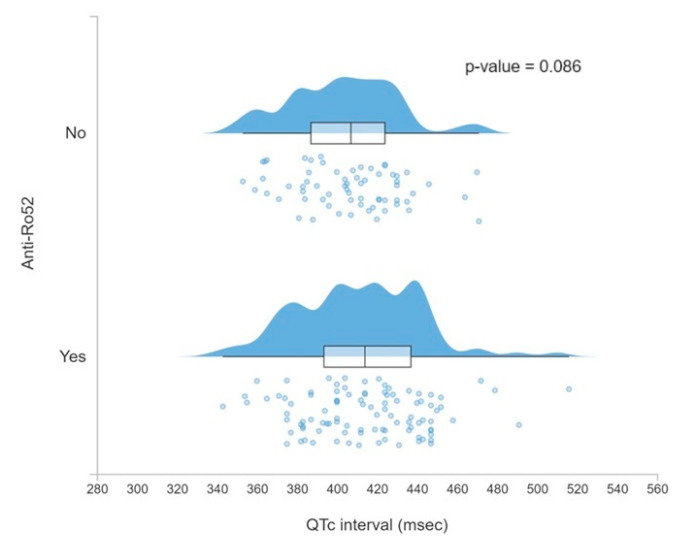
Population density according to QTc interval segregated by the presence of anti-Ro52.

Among anti-Ro52-positive patients, more cardiac rhythm disorders were found (relative risk (RR) = 2.01, 95% CI 1.20–3.37), specifically QTc interval prolongation (RR = 4.25, 95% CI 1.55–11.62), with a longer average QTc duration close to statistical significance (*p* = 0.086) as can be seen in Figure 3.

After performing a bivariate regression analysis, variables significantly associated with the presence of any cardiac rhythm disorder were entered into a multivariate logistic regression model. DM, chronic respiratory disease, and anti-Ro52 positivity were independent predictors for developing cardiac rhythm disorder (Table 2).

As shown in Table 2, each variable was associated with one or more specific cardiac rhythm disorders, with intraventricular conduction disorders predominating in most cases. In our study, anti-Ro52 positivity increased only the risk of QTc prolongation but not the risk of AV conduction disturbances.

### 3.3. AV Conduction Disorders

The analysis was repeated with AV conduction disturbances, and a statistically significant relationship was found in the bivariate analysis with the existence of CKD, SLE, and exposure to β-blockers or biological drugs. Antimalarial use was close to significance (*p* = 0.085). Almost 90% of SLE patients were exposed to antimalarials. When evaluating the relationship between antimalarials and AV conduction disorders in patients without SLE, statistical significance was not reached (*p* = 0.085), so we considered the existence of collinearity between SLE and antimalarial intake. No significant association was found with strong-positivity or time of exposure to anti-Ro52 antibodies. After multivariate logistic regression analysis, the existence of CKD, SLE, and exposure to biologic drugs were the only ones that maintained significance (Table 3). Time from diagnosis in SLE patients was more extended in those with AVB without reaching statistical significance (13.4 vs. 10.6 years, *p* = 0.485). No statistically significant association was found between anti-Ro52 positivity and the presence of AV conduction disorders (RR = 1.53, 95% CI 0.41–5.69).

### 3.4. QTc Prolongation

**Figure 4:**
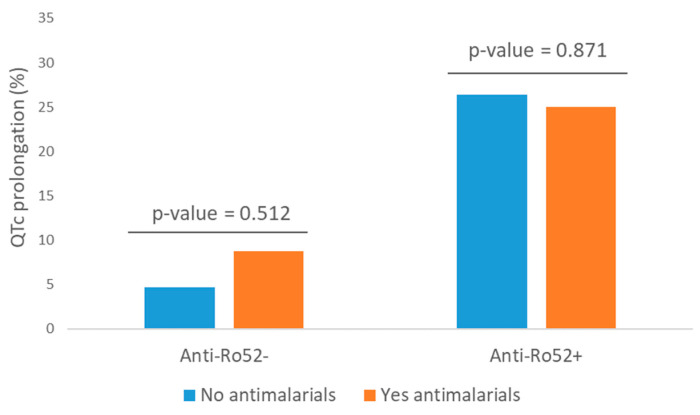
QTc prolongation segregated by the presence of anti-Ro52 and active antimalarial treatment.

Variables presenting a statistically significant relationship with the presence of QTc prolongation in the bivariate analysis are shown in Table 3. Despite the potential risk of antimalarials producing prolonged QTc [34], no statistical association was found in our study (*p* = 0.612; RR = 1.18, 95% CI 0.62–2.26) (Figure 4). We also did not find statistical significance when studying this relationship in the subgroup of patients over 65 years. Exposure to tricyclic antidepressants or antipsychotics was not associated with a higher risk of QTc prolongation (*p* = 1.000 and 0.587, respectively), nor cumulative time of exposure to anti-Ro52 antibodies. QTc prolongation was more frequent in patients with strong anti-Ro52 positivity compared to negative or not-strong positivity (27.8% vs. 10.5%, RR = 2.64, 95% CI 1.29–5.38, *p* = 0.004). Collinearity was detected between anti-Ro60 and anti-Ro52 antibodies; 86% of patients with anti-Ro60 were positive for anti-Ro52 (77% in the reverse situation), and when we evaluated their role selectively in the anti-Ro52+ population, the statistical association disappeared (*p* > 0.05 for all of them). Therefore, they were not incorporated into the multivariate regression model, in which only DM and anti-Ro52 positivity remained independent risk predictors (Table 3).

## 4. Discussion

To our knowledge, this is the most extensive published series to evaluate cardiac rhythm disorders in anti-Ro52-positive patients in our country. We found that one third of the patients included in our study (57 out of 167) had some cardiac rhythm disorder. Regarding the abnormalities associated with anti-Ro/SSA or anti-La/SSB positivity, we observed that anti-Ro52 antibodies were the only ones significantly associated with the risk of cardiac rhythm disorders (two times higher than in the seronegative cohort), specifically prolongation of the QT interval [35]. In all of them, the alteration found was asymptomatic and mild, except for one patient. We did not observe a higher risk of electrocardiographic alterations associated with anti-Ro52 antibodies in patients with acquired myocardial injury, so our study did not confirm the cardiomyocyte apoptosis hypothesis in adult patients.

We did not detect any interaction between positivity, titer strength, or time of exposure to anti-Ro52 and AV conduction disorders, although they were quite less frequent than QTc interval prolongation (6% vs. 18%) and could have influenced the lack of association. AV conduction disorders seemed to depend significantly on the existence of CKD, SLE, or active biological treatment, not on the anti-Ro52 status or active exposure to corticosteroids, antimalarials, or negative chronotropic drugs. Altogether, it could be hypothesized that in our series, AV conduction disorders were more mediated by chronic microvascular damage and subsequent fibrosis of the conduction tissue due to the associated underlying diseases. We found a non-significant increase in the time from diagnosis of SLE in patients with AVB. A bigger sample size and the use of chronic damage scales in SLE would better confirm such a hypothesis.

The association between anti-Ro52 positivity and QTc prolongation is much more evident and affects one in four patients, in line with previous reports. The significance of QTc interval prolongation lies in the potential risk of presenting polymorphic ventricular tachycardia, known as Torsade de pointes, or sudden death, although this complication is infrequent and appears when QTc > 500 ms [36]. We did not observe an enhancement effect of double (anti-Ro60 and anti-Ro52) antibody positivity or the time of exposure to them. We noted that strong anti-Ro52 titers increased the frequency of prolonged QTc without reaching statistical significance in multivariate analysis, maybe due to sample size. No association with any specific SAD was observed either [16]. This absence of association is reaffirmed by the description of QTc abnormalities and the development of Torsade de pointes in asymptomatic anti-Ro52 carriers [20]. The risk of prolongation of QTc interval associated with anti-Ro52 seems to be increased in diabetic patients [37]. Apart from genetic predisposition in some patients, drugs are the leading cause of acquired QT interval prolongation [38], including antiarrhythmic drugs, some antibiotics (macrolides, quinolones) or psychiatric medications, among others, although their distribution in our series was balanced between anti-Ro52-positive and negative patients and did not influence the probability of finding QTc prolongation. When evaluating antimalarials, which are widely used in patients with SADs and associated with long QTc intervals, we did not find a potentiating effect on the risk of prolonged QTc in our series, so we suggest that they can be continued with caution and close monitoring in cases of mild prolongation of the baseline QTc interval, to anticipate situations of greater arrhythmic risk (QTc > 500 ms).

The relationship between cardiac rhythm disorders in adult patients and the presence of anti-Ro/SSA antibodies, well established for the risk of heart disease and congenital AVB in the context of neonatal lupus, is currently under debate. They have also been associated with anti-La/SSB antibodies, frequently coexisting with anti-Ro/SSA; however, with a lesser degree of evidence [8]. Since Scott et al. [39] first asked why these autoantibodies affected the fetal heart and not the maternal heart, there have been numerous reports of isolated cases of cardiac rhythm disorders associated with anti-Ro/SSA and also some tiny series (usually with less than 50 cases carrying these autoantibodies), either in the general population or in patients with a specific SAD (SLE, SS, dermatomyositis, scleroderma), where the analysis of the association between rhythm disorders and anti-Ro/SSA has yielded disparate conclusions. In two of the most recently published articles in 2021, Villuendas et al. [26] found no association between positivity against anti-Ro/SSA and alterations in EKG intervals (49 anti-Ro+ cases). Lazzerini et al. [3], in a population-based series of U.S. Veterans where anti-Ro/SSA positivity was screened (612 anti-Ro+ cases), found that the presence of these autoantibodies was associated with the risk of presenting QTc interval prolongation.

It seems evident that the prevalence of advanced cardiac rhythm disorders in adult anti-Ro/SSA carriers with or without an associated SAD is a highly infrequent complication, and therefore, small series are not sufficiently powered to demonstrate a causal relationship. This sample size problem was one of the determinants of our study design: to increase the sample size by capturing a more significant number of seropositive patients at the expense of a lower homogeneity when working with the hypothesis that electrographic abnormalities associated with anti-Ro52 were independent of the underlying SAD.

Our study has several limitations. On one hand, the cross-sectional design did not allow us to predict the long-term clinical significance of mild rhythm disorders found. Also, our study could be affected by a selection bias as the recruitment of anti-Ro52-negative patients did not reach the expected sample size, and the prevalence of prolonged QTc in this population could have been underestimated. The lack of observed association between anti-Ro antibodies and EKG disorders in some studies is probably due to insufficient recruitment of anti-Ro52 carriers. By increasing the number of these patients, we found a clear association with QTc prolongation, consistent with Lazzerini et al.’s findings, so these recruitment limitations should not invalidate our conclusions. As various SADs were included in the study, we could not compare disease activity or accrual damage well, as homogeneous standardized scales were unavailable. That is why the influence of disease activity or accrual damage on the development of cardiac rhythm disorders could be underestimated in our study. Dichotomous drug intake assessment did not let us confirm a dose-dependent effect of drugs on cardiac rhythm disorders. As cardiac evaluation was made exclusively by a 12-lead EKG, asymptomatic significant structural cardiac disease or paroxysmal rhythm disorders could not be excluded entirely in our patients. Finally, given that the reversibility of rhythm disturbances in anti-Ro52 patients treated with immunosuppressants has been described and that new risk factors for cardiac rhythm disturbances may be added (e.g., drugs, comorbidities), a long-term follow-up to confirm the impact of our findings is mandatory.

The strengths of this study include the following. As cardiac rhythm abnormalities depend more on antibody status than on the associated SAD, not restricting the study to a single SAD (e.g., SLE) let us increase the sample size concerning previous series, which is critical for the strength of statistical analysis and identifying associations. Antibody status in SAD adult patients is only another risk factor for cardiac rhythm disorders. The consideration of age, medications, and comorbidities, and the performance of multivariate regression analysis allowed us to estimate the impact of this interaction better. Finally, excluding seropositive patients without an associated SAD (asymptomatic carriers) lets us better ensure if a chronic inflammatory status, as chronic systemic inflammation has been associated with microvascular damage, may also play a role in developing cardiac rhythm disorders in these patients.

As future lines of research, long-term follow-up of patients is crucial to assess the clinical significance of mild asymptomatic QTc interval prolongation observed in this cross-sectional study. Systematic echocardiographic evaluation, monitoring of strength and antibody status, continuous pharmacological evaluation with a focus on dosing, and a balanced cohort sample size will help to overcome the limitations of the present study. Also, further research focusing on antimalarials and their association with cardiac rhythm disorders in anti-Ro52 patients with SADs could be very relevant in clinical practice.

## 5. Conclusions

This daily clinical practice series of adults with SADs showed that approximately one third of patients had rhythm disorders, especially intraventricular and QTc prolongation. The probability of finding QTc prolongation in patients with anti-Ro/SSA antibodies is specific to anti-Ro52 antibodies, and its strong positivity could increase the risk. We detected no relationship with the strength of the titers, the duration of positivity, or the existence of double or triple positivity. Nor is it related to negative chronotropic drugs, antimalarials, or active corticosteroid exposure. Its effect can be potentiated by diabetes mellitus and QTc interval prolonging drugs. As for AV conduction disorders, which are much less frequent, we found no association in our study with anti-Ro52 antibodies, although accumulated clinical experience supports it, with SLE as the possibly most related SAD. A generalized screening by surface EKG in patients with anti-Ro52 antibodies does not seem justified for early AV conduction disturbances, although it may be advisable to detect long QT syndrome, especially in patients with anti-Ro52 strong-positivity, diabetes, and exposure to risky drugs. A prospective follow-up of patients with anti-Ro52 antibodies in whom electrocardiographic abnormalities have been identified would help better determine the long-term clinical significance of these findings.

## Figures and Tables

**Table 1:** Baseline features in the overall series and according to anti-Ro52 positivity.

	Overall Series (*n* = 167)	Anti-Ro52+ (*n* = 101)	Anti-Ro52− (*n* = 66)	*p*-Value
Age years (SD)	59.19 (12.8)	60.21 (13.4)	57.62 (11.7)	0.202
Female sex *n* (%)	140 (83.8)	89 (88.1)	51 (77.3)	0.063
Smoker (active or former) *n* (%)	60 (35.9)	33 (32.7)	27 (40.9)	0.278
Hypertension *n* (%)	53 (31.7)	31 (30.7)	22 (33.3)	0.720
Diabetes mellitus *n* (%)	14 (8.4)	7 (6.9)	7 (10.6)	0.402
Dyslipidemia *n* (%)	56 (33.5)	27 (26.7)	29 (43.9)	0.021 *
Heart disease *n* (%)	16 (9.6)	9 (8.9)	7 (10.6)	0.716
Respiratory disease *n* (%)	33 (19.8)	19 (18.8)	14 (21.4)	0.703
CKD *n* (%)	12 (7.2)	11 (10.9)	1 (1.5)	0.022 *
Electrolyte disorders *n* (%)	4 (2.4)	4 (4.0)	0 (0.0)	0.102
Thyroid disease *n* (%)	41 (24.6)	27 (26.7)	14 (21.2)	0.418
Β-blockers *n* (%)	15 (9.0)	11 (10.9)	4 (6.1)	0.286
Non-dihydropyridine calcium antagonists *n* (%)	3 (1.8)	1 (1.0)	2 (3.0)	0.332
Other drugs studied *n* (%)	15 (9.0)	7 (6.9)	8 (12.1)	0.251
SAD *n* (%) - pSS - SLE and cutaneous lupus - RA - Scleroderma - Other SAD	58 (34.7) 41 (24.6) 38 (22.8) 10 (6.0) 39 (23.4)	52 (51.5) 25 (24.8) 12 (11.9) 7 (6.9) 21 (20.8)	6 (9.1) 16 (24.2) 26 (39.4) 3 (4.5) 18 (27.3)	<0.001 * 0.940 <0.001 * 0.525 0.333
Time from diagnosis years (IQR)	7 (11)	6 (11)	10 (13)	0.141
Corticosteroids *n* (%)	50 (29.9)	23 (22.8)	27 (40.9)	0.012 *
Antimalarials *n* (%)	71 (42.5)	48 (47.5)	23 (34.8)	0.105
Immunosuppressants *n* (%)	56 (33.5)	22 (21.8)	34 (51.5)	<0.001 *
Biologic drugs *n* (%)	12 (7.2)	5 (5.0)	7 (10.6)	0.166
Rhythm disorders *n* (%)	57 (34.1)	43 (42.6)	14 (21.2)	0.004 *
- Sinoatrial conduction disorders *n* (%)	5 (3.0)	4 (4.0)	1 (1.5)	0.365
- AV conduction disorders *n* (%)	10 (6.0)	7 (6.9)	3 (4.5)	0.525
- Intraventricular conduction disorders *n* (%)	23 (13.8)	14 (13.9)	9 (13.6)	0.967
- QTc prolongation *n* (%)	30 (18.0)	26 (25.7)	4 (6.1)	0.001 *
- Supraventricular tachyarrhythmias *n* (%)	6 (3.6)	3 (3.0)	3 (4.5)	0.593
- Ventricular tachyarrhythmias *n* (%)	0 (0)	0 (0)	0 (0)	
RR interval ms (SD)	860 (157)	843 (140)	881 (178)	0.145
PQ interval ms (IQR)	162 (40)	162 (40)	163 (40)	0.890
QRS interval ms (IQR)	80 (30)	79 (20)	82 (20)	0.217
QTc interval ms (SD)	412 (29)	415 (31)	407 (26)	0.086

* Statistical significance (*p*-value < 0.05). SD: standard deviation, CKD: chronic kidney disease, SAD: systemic autoimmune disease, IQR: interquartile range, pSS: primary Sjögren’s syndrome, SLE: systemic lupus erythematosus, RA: rheumatoid arthritis, AV: atrioventricular QTc: corrected QT interval, ms: milliseconds.

**Table 2:** Bivariate and multivariate analysis for the development of any cardiac rhythm disorder. Logistic regression.

	Bivariate Analysis (*p*-Value)	Specific Cardiac Rhythm Disorder (*p*-Value)	Multivariate Regression (*p*-Value)	Multivariate Regression OR (CI 95%)
Age	0.010	Intraventricular (0.001) QTc prolongation (0.009)	0.516	1.01 (0.98–1.04)
HTN	0.017	Intraventricular (0.001) Tachyarrhythmias (0.026)	0.289	1.55 (0.68–3.53)
DM	0.005	Intraventricular (0.020) QTc prolongation (0.017)	0.009 *	6.00 (1.56–23.11)
Chronic respiratory disease	0.049	Intraventricular (0.015)	0.043 *	2.45 (1.03–5.84)
CKD	0.021	AV disorder (0.001) QTc prolongation (0.036)	0.227	2.32 (0.59–9.11)
Anti-Ro52+	0.005	QTc prolongation (0.003)	0.004 *	3.19 (1.43–7.13)

* Statistically significant in multivariate regression analysis (*p*-value < 0.05). HTN: arterial hypertension, DM: diabetes mellitus, CKD: chronic kidney disease, AV: atrioventricular.

**Table 3:** Bivariate and multivariate analysis for the development of any AV conduction disorder and QTc prolongation. Logistic regression.

	Bivariate Analysis (*p*-Value)	Multivariate Regression (*p*-Value)	Multivariate Regression OR (CI 95%)
**AV conduction disorders**			
CKD	0.001	0.019 *	8.82 (1.42–54.67)
β-blockers	0.029	0.377	2.47 (0.33–18.40)
SLE	0.046	0.044 *	5.04 (1.05–24.27)
Biologic drugs	0.011	0.004 *	15.08 (2.32–97.89)
Antimalarials	0.085		
**QTc prolongation**			
Age	0.009	0.083	1.03 (1.00–1.07)
DM	0.017	0.035 *	4.48 (1.11–17.12)
CKD	0.036	0.522	1.56 (0.40–6.12)
Anti-Ro52+	0.003	0.036 *	6.05 (1.11–20.16)
Anti-Ro52 strong-positivity	0.005	0.542	1.40 (0.48–4.05)
Anti-Ro60+	0.022		

* Statistical significance in multivariate regression analysis (*p*-value < 0.05). CKD: chronic kidney disease, SLE: systemic lupus erythematous, DM: diabetes mellitus.

## Data Availability

The data presented in this study are available upon reasonable request to the corresponding author due to the permission conditions of the Investigation Ethics Committee of the Hospital Clínico Universitario Health Area.

## Supplementary Materials

The following supporting information can be downloaded at: https://www.mdpi.com/article/10.3390/jcm13123510/s1, Table S1. Summary of published cases associating anti-Ro/SSA and AV block in adults; Table S2. Summary of published studies assessing the association between anti-Ro/SSA antibodies and QTc prolongation; Table S3. Variables of study; Table S4. Anti-Ro60, anti-Ro52 and anti-La/SSB antibodies: positivity, titer strength, and time since first positivity [3,15,17,18,19,21,25,26,27,35,40,41,42,43,44,45,46,47,48,49,50,51,52,53,54,55,56,57,58,59,60,61,62,63,64,65,66,67,68,69,70].

## Abbreviation List

AV: atrioventricular, AVB: atrioventricular block, CI: confidence interval, CKD: chronic kidney disease, DM: diabetes mellitus, EKG: electrocardiogram, HTN: hypertension, IQR: interquartile range, kDa: kilodaltons, ms: milliseconds, pSS: primary Sjögren’s syndrome, QTc: QT interval corrected for heart rate, RA: rheumatoid arthritis, RR: relative risk, SAD: systemic autoimmune diseases, SD: standard deviation, SLE: systemic lupus erythematosus, χ^2^: chi-square test.
