# Supplementary material for: Should Physicians Be Aware of Rhythm Disturbances in Adults with Systemic Autoimmune Diseases and Anti-Ro52 Antibodies? A Cross-Sectional Study
Source: J Clin Med. 2024 Jun 15;13(12):3510. doi: 10.3390/jcm13123510 (PMC11204400; doi:10.3390/jcm13123510)
Supplement: Supplementary file 1 [file jcm-13-03510-s001.zip › jcm-3001340-supplementary.pdf]

SUPPLEMENTARY MATERIAL

Table S1. Summary of published cases associating anti-Ro/SSA and AV block in adults.

Publication (year)	Degree AVB	Sex	Age	SAD	Symptoms	Treatment	Outcome
Lazzerini (2023) [1]	34 AVB of unknown cause: 18 patients (52.9%) were anti-Ro+<br>Corticosteroid improved AVB in anti-Ro+ but not in anti-Ro-						
Akuka (2022) [2]	A large cross-sectional study (17231 anti-Ro/anti-La+ and 84368 controls) found a significant increase in the risk of AVB and other cardiac rhythm disorders in anti-Ro+						
Lazzerini (2022) [3]	2nd degree Möbitz I-II	M	65	No SAD	Dyspnea<br>Presyncope	Corticosteroids<br>HCQ	1st degree block
Jobling (2018) [4]	3rd degree	F	44	pSS	Dyspnea	Pacemaker	Asymptomatic
Tam (2018) [5]	3rd degree	F	57	pSS	Dyspnea<br>Presyncope	HCQ<br>Pacemaker	Asymptomatic
Lazzerini (2015) [6]	3rd degree	F	29	No SAD	Chest pain<br>Syncope	Corticosteroids<br>Azathioprine	Resolution
Saribayev (2014) [7]	2nd degree Möbitz I	M	77	SLE	Dyspnea<br>Edemas	Corticosteroids	1st degree block
Villuendas (2014) [8]	Pacemaker for AVB of unknown cause:<br>2 cases (10.5%) were anti-Ro+						
Santos-Pardo (2013) [9]	3rd degree	F	26	No SAD	Syncope	Corticosteroids<br>Azathioprine	Resolution
Sung (2011) [10]	2nd (2:1) and 3rd degree	F	49	pSS	Dyspnea	Pacemaker	Asymptomatic
Adami (2009) [11]	3rd degree	F	37	USAD	Sudden cardiac arrest	Advanced CPR	Persistent vegetative state
Arce-Salinas (2009) [12]	3rd degree	F	19	SLE	Seizures	Pacemaker<br>Corticosteroids<br>Azathioprine	SAD symptoms
Yavasoglu (2007) [13]	2nd degree Mobitz I	F	59	SLE	Tiredness	Corticosteroids	Resolution
Lim (2005) [14]	3rd degree	F	59	SLE	Dyspnea	Corticosteroids<br>Pacemaker	Resolution
Liautaud (2005) [15]	2nd degree Mobitz I	M	45	SLE	No	Corticosteroids	1st degree block
Lodde (2005) [16]	No association between anti-Ro+ and PR prolongation in pSS patients (yes with anti-La/SSB)						
Comín-Colet (2001) [17]	2nd degree Mobitz II	F	40	SLE	Presyncope	Corticosteroids<br>HCQ	Resolution
Baumgart (1998) [18]	3rd degree	F	76	pSS	Syncope	Corticosteroids<br>HCQ	Resolution
Lee (1996) [19]	3rd degree	F	39	pSS	Presyncope	Pacemaker	SAD symptoms
Mevorach (1993) [20]	3rd degree	F	29	SLE	Syncope	Pacemaker	SAD symptoms
Martínez-Costa (1991) [21]	3rd degree	F	39	SLE	Syncope	Pacemaker	-
Logar (1990) [22]	8 anti-Ro+ patients presented cardiac conduction disorders<br>(1st degree AVB, RBB and LAFB)						
Bilazarian (1989) [23]	3rd degree	F	42	SLE	Presyncope	Corticosteroids	Resolution

Maier (1987) [24]	3rd degree	F	27	SLE	Chest pain	Temporary pacemaker	Asymptomatic
Behan (1986) [25]	RBBB	F	59	DM	Weakness<br>Muscle pain	Corticosteroids	CHF<br>Dead

AVB: atrioventricular block, **SAD:** systemic autoimmune disease, **F**: female sex, **M**: male sex, **HCQ**: hydroxychloroquine, **pSS**: primary Sjögren's syndrome, **SLE**: systemic lupus erythematosus, **USAD:** undifferentiated SAD, **CPR:** cardiopulmonary resuscitation, **RBBB**: right bundle branch block, **LAFB**: left anterior fascicular block, **DM**: dermatomyositis, **CHF**: congestive heart failure.

SUPPLEMENTARY MATERIAL Table S2. Summary of published studies assessing the association between anti-Ro/SSA antibodies and QTc prolongation.

Study (year)	Disease	Sample size	Anti-Ro+ (%)	Test	Results
Hu (2022) [26]	SLE	299	132 (44.1)	EKG	Anti-Ro+ was the most important independent variable in a predictive machine learning model for QT prolongation
Lazzerini (2021) [27]	No SAD	7339	612 (8.3)	EKG	QTc prolongation in anti-Ro+ Synergy with other RF
Villuendas (2021) [28]	SLE	145	49 (32)	EKG<br>ECO<br>EKG-Holter 24 h	No significant differences between anti-Ro+ and -
Mostafavi (2020) [29]	SLE	150	55 (36.7)	EKG	Increased % of anti-Ro/SSA+ if QTc prolongation
Lazzerini (2016) [30]	TdP	25	15 (60)	EKG	QTc mean higher in anti-Ro+. Higher prevalence of anti-Ro+ in TdP patients
Perez-García (2016) [31]	SLE	66	-	EKG	Positive linear association between anti-Ro52 levels and QTc duration
Tufan (2016) [32]	SAD	76	15 (19.7)	EKG	Tpeak-Tend duration and Tpeak-Tend duration/QT ratio was higher in anti-Ro52+. Strong correlation with its titers
Pisoni (2015) [33]	SAD	73	55 (75)	EKG	More QTc prolongation in anti-Ro+ (p-value=0.05)
Sham (2015) [34]	SLE	200	100 (50)	EKG	QTc mean higher in anti-Ro+
Massie (2014) [35]	SS	689	148 (21.5)	EKG	No difference in QTc between anti-Ro+ and -
Nomura (2014) [36]	SLE	91	43 (47.7)	EKG	Increased % of anti-Ro/SSA+ if QTc prolongation (NS)
Bourré-Tessier (2011) [37]	SLE	150 + 278	57 (38.0) + 113(41.0)	EKG	Association between anti-Ro+ and QTc prolongation
Lazzerini (2011) [38]	SAD	49	25 (51)	EKG	Association between anti-Ro+ and QTc prolongation Relationship with anti-Ro titers
Lazzerini (2007) [39]	SAD	46	26 (56.5)	EKG-Holter 24h	In anti-Ro+, higher frequency of QTc prolongation and complex ventricular arrhythmias
Lazzerini (2004) [40]	SAD	57	31 (54.3)	EKG	Association between anti-Ro+ and QTc prolongation
Logar (1990) [22]	SLE	67	36 (53,7)	EKG and/or ECO	Association between anti-Ro+ and conduction disorders or myocarditis
Behan (1987) [41]	Polymyositis	55	33 (60)	EKG	Higher anti-Ro+ proportion in the group with rhythm disorders

SAD: systemic autoimmune disease, **EKG**: electrocardiogram, **QTc**: corrected QT interval, **RF**: risk factors for QTc prolongation, **SLE**: systemic lupus erythematosus, **ECO**: echocardiogram, **TdP**: torsade de pointes, **SS**: systemic sclerosis, **NS**: P-value>0.05, **AVB**: atrioventricular block.

SUPPLEMENTARY MATERIAL Table S3. Variables of study.

**Epidemiological variables**
Age
Sex
Race
**Family and personal history**
Family history (hereditary heart disease, progenitors with SAD)
Smoking
Alcoholism
Drugs of abuse
Hypertension
Diabetes mellitus
Dyslipidemia
Heart disease (ischemic, valvular, etc.)
Chronic respiratory diseases
Chronic kidney disease (GFR<60 ml/min)
Electrolytic alterations
Thyroid pathology
Deposit disease
Cancer
**Information about SAD**
Type of SAD
Time since diagnosis
**Active drug treatments**
Corticosteroids
Antimalarials
Immunosuppressants
Biologics drugs
β-blockers
Non-dihydropyridine Ca antagonists
Antiarrhythmics
Tricyclic antidepressants
Antipsychotics
Antihistamines
Conduction altering drugs
**Immunological profile**
Anti-Ro60 (positivity, strength of positivity and time since 1st positivity)
Anti-Ro52 (positivity, strength of positivity and time since 1st positivity)
Anti-La (positivity, strength of positivity and time since 1st positivity)
Anti-ANA
Anti-native DNA
Anti-RNP
Anti-SM
Anti-Jo1
Anti-histone
Anti-Scl70
Anti-centromere
Rheumatoid factor
Serum complement levels

SUPPLEMENTARY MATERIAL Table S4. Anti-Ro60, anti-Ro52 and anti-La/SSB antibodies: positivity, titer strength, and time since first positivity.

	**Positivity for antibodies (%)**	**Strong antibody positivity (%)**	**Years since first positivity (IQR)**
Anti-Ro60	90 (53,9)	62 (37,1)	6 (10)
Anti-Ro52	101 (60,5)	72 (43,1)	6 (9)
Anti-La	45 (26,9)	24 (14,4)	6 (11)

IQR: interquartile range.

REFERENCES FOR SUPPLEMENTARY MATERIAL

1. Lazzerini, P.E.; Murthy Ginjupalli, V.K.; Srivastava, U.; Bertolozzi, I.; Bacarelli, M.R.; Verrengia, D.; Salvini, V.; Accioli, R.; Carbone, S.F.; Santoro, A.; et al. Anti-Ro/SSA Antibodies Blocking Calcium Channels as a Potentially Reversible Cause of Atrioventricular Block in Adults. *JACC Clin Electrophysiol* **2023**, *9*, 1631–1648, doi:10.1016/j.jacep.2023.03.007.

2. Akuka, A.; Ben-Shabat, N.; Watad, A.; Tsur, A.M.; Ehrenberg, S.; McGonagle, D.; Comaneshter, D.; Beinart, R.; Cohen, A.D.; Amital, H. Association of Anti-Ro Seropositivity with Cardiac Rhythm and Conduction Disturbances. *Eur Heart J* **2022**, *43*, 4912–4919, doi:10.1093/eurheartj/ehac516.

3. Lazzerini, P.E.; Salvini, V.; Srivastava, U.; Ginjupalli, V.K.M.; Santoro, A.; Bertolozzi, I.; Accioli, R.; Laghi-Pasini, F.; Boutjdir, M.; Capecchi, P.L. Anti-Ca v 1.2 Antibody–Induced Atrioventricular Block as a Novel Form in the Adult: Long-Term Pacemaker-Sparing Activity of Hydroxychloroquine. *Circ Arrhythm Electrophysiol* **2022**, *15*, doi:10.1161/CIRCEP.122.011042.

4. Jobling, K.; Rajabally, H.; Ng, W.-F. Anti-Ro Antibodies and Complete Heart Block in Adults with Sjögren's Syndrome. *Eur J Rheumatol* **2018**, *5*, 194–196, doi:10.5152/eurjrheum.2018.18019.

5. Tam, W.K. Association of Anti-Ro/Sjögren's Syndrome Type A Antibodies and Complete Atrioventricular Block in an Adult With Sjögren's Syndrome. *Arch Rheumatol* **2018**, *33*, 225–229, doi:10.5606/ArchRheumatol.2018.6492.

6. Lazzerini, P.E.; Brucato, A.; Capecchi, P.L.; Baldi, L.; Bacarelli, M.R.; Nucci, C.; Moscadelli, V.; Morozzi, G.; Boutjdir, M.; Laghi-Pasini, F. Isolated Atrioventricular Block of Unknown Origin in the Adult and Autoimmunity: Diagnostic and Therapeutic Considerations Exemplified by 3 Anti-Ro/SSA–Associated Cases. *HeartRhythm Case Rep* **2015**, *1*, 293–299, doi:10.1016/j.hrcr.2015.03.019.

7. Saribayev, M.; Tufan, F.; Oz, F.; Erer, B.; Ozpolat, T.; Ozturk, G.B.; Akin, S.; Saka, B.; Erten, N.; Tascioglu, C.; et al. Corticosteroid Treatment Normalizes QTc Prolongation and Improves Heart Block in an Elderly Patient with Anti-Ro-Positive Systemic Lupus Erythematosus. *Aging Clin Exp Res* **2014**, *26*, 337–339, doi:10.1007/s40520-013-0168-9.

8. Villuendas, R.; Olivé, A.; Juncà, G.; Salvador, I.; Martínez-Morillo, M.; Santos-Pardo, I.; Pereferrer, D.; Zamora, E.; Bayes-Genis, A. Autoimmunity and Atrioventricular Block of Unknown Etiology in Adults. *J Am Coll Cardiol* **2014**, *63*, 1335–1336, doi:10.1016/j.jacc.2013.10.086.

9. Santos-Pardo, I.; Martínez-Morillo, M.; Villuendas, R.; Bayes-Genis, A. Anti-Ro Antibodies and Reversible Atrioventricular Block. *New England Journal of Medicine* **2013**, *368*, 2335–2337, doi:10.1056/NEJMc1300484.

10. Sung, M.J.; Park, S.-H.; Kim, S.-K.; Lee, Y.-S.; Park, C.-Y.; Choe, J.-Y. Complete Atrioventricular Block in Adult Sjögren's Syndrome with Anti-Ro Autoantibody. *Korean J Intern Med* **2011**, *26*, 213, doi:10.3904/kjim.2011.26.2.213.

11. Adami, M.; Nardin, M.; Morello, E.; Crivellaro, C.; Wiedermann, C.J. Fatal Cardiac Arrest in an Adult Patient with Euthyroid Anti-SSA/Ro-Positive Connective Tissue Disease: A Case Report. *Int Arch Med* **2009**, *2*, 15, doi:10.1186/1755-7682-2-15.

12. Arce-Salinas, C.A.; Carmona-Escamilla, M.A.; Rodríguez-García, F. Complete Atrioventricular Block as Initial Manifestation of Systemic Lupus Erythematosus. *Clin Exp Rheumatol* **2009**, *27*, 344–346.

13. Yavasoglu, I.; Kadikoylu, G.; Bolaman, Z. Adult Systemic Lupus Erythematosus and Secondary Atrioventricular Block. *J Electrocardiol* **2007**, *40*, S26–S27, doi:10.1016/j.jelectrocard.2007.03.193.

14. Lim, L.; Joshua, F. Resolution of Complete Heart Block after Prednisolone in a Patient with Systemic Lupus Erythematosus. *Lupus* **2005**, *14*, 561–563, doi:10.1191/0961203305lu2155xx.

15. Liautaud, S.; Khan, A.J.; Nalamasu, S.R.; Tan, I.J.; Onwuanyi, A.E. Variable Atrioventricular Block in Systemic Lupus Erythematosus. *Clin Rheumatol* **2005**, *24*, 162–165, doi:10.1007/s10067-004-0995-3.

16. Lodde, B.M.; Sankar, V.; Kok, M.R.; Leakan, R.A.; Tak, P.P.; Pillemer, S.R. Adult Heart Block Is Associated with Disease Activity in Primary Sjögren's Syndrome. *Scand J Rheumatol* **2005**, *34*, 383–386, doi:10.1080/03009740510026661.

17. Comín-Colet, J.; Sánchez-Corral, M.A.; Alegre-Sancho, J.J.; Valverde, J.; López-Gómez, D.; Sabaté, X.; Juan-Mas, A.; Esplugas, E. Complete Heart Block in an Adult with Systemic Lupus Erythematosus and Recent Onset of Hydroxychloroquine Therapy. *Lupus* **2001**, *10*, 59–62, doi:10.1191/096120301673172543.

18. Baumgart, D.C.; Gerl, H.; Dorner, T. Complete Heart Block Caused by Primary Sjogren's Syndrome and Hypopituitarism. *Ann Rheum Dis* **1998**, *57*, 635–635, doi:10.1136/ard.57.10.635.

19. Lee, L.A.; Pickrell, M.B.; Reichlin, M. Development of Complete Heart Block in an Adult Patient with Sjögren's Syndrome and Anti-Ro/SS-A Autoantibodies. *Arthritis Rheum* **1996**, *39*, 1427–1429, doi:10.1002/art.1780390825.

20. Mevorach, D.; Raz, E.; Shalev, O.; Steiner, I.; Ben-Chetrit, E. Complete Heart Block and Seizures in an Adult with Systemic Lupus Erythematosus: A Possible Pathophysiologic Role for Anti-SS-A/Ro and Anti-SS-B/La Autoantibodies. *Arthritis Rheum* **1993**, *36*, 259–262, doi:10.1002/art.1780360219.

21. Martinez-Costa, X.; Ordi, J.; Barberá, J.; Selva, A.; Bosch, J.; Vilardell, M. High Grade Atrioventricular Heart Block in 2 Adults with Systemic Lupus Erythematosus. *J Rheumatol* **1991**, *18*, 1926–1928.

22. Logar, D.; Kveder, T.; Rozman, B.; Dobovisek, J. Possible Association between Anti-Ro Antibodies and Myocarditis or Cardiac Conduction Defects in Adultswith Systemic Lupus Erythematosus. *Ann Rheum Dis* **1990**, *49*, 627–629, doi:10.1136/ard.49.8.627.

23. Bilazarian, S.D.; Taylor, A.J.; Brezinski, D.; Hochberg, M.C.; Guarnieri, T.; Provost, T.T. High-Grade Atrioventricular Heart Block in an Adult with Systemic Lupus Erythematosus:

The Association of Nuclear RNP (U1 RNP) Antibodies, a Case Report, and Review of the Literature. *Arthritis Rheum* **1989**, *32*, 1170–1174, doi:10.1002/anr.1780320918.

24. Maier, W.P. Complete Heart Block as the Initial Manifestation of Systemic Lupus Erythematosus. *Arch Intern Med* **1987**, *147*, 170, doi:10.1001/archinte.1987.00370010168034.

25. Behan, W.M.; Aitchison, M.; Behan, P.O. Pathogenesis of Heart Block in a Fatal Case of Dermatomyositis. *Br Heart J* **1986**, *56*, 479–482, doi:10.1136/hrt.56.5.479.

26. Hu, Z.; Wu, L.; Lin, Z.; Liu, X.; Zhao, C.; Wu, Z. Prevalence and Associated Factors of Electrocardiogram Abnormalities in Patients With Systemic Lupus Erythematosus: A Machine Learning Study. *Arthritis Care Res (Hoboken)* **2022**, *74*, 1640–1648, doi:10.1002/acr.24612.

27. Lazzerini, P.E.; Cevenini, G.; Qu, Y.S.; Fabris, F.; El-Sherif, N.; Acampa, M.; Cartocci, A.; Laghi-Pasini, F.; Capecchi, P.L.; Boutjdir, M.; et al. Risk of QTc Interval Prolongation Associated With Circulating Anti-Ro/SSA Antibodies Among US Veterans: An Observational Cohort Study. *J Am Heart Assoc* **2021**, *10*, doi:10.1161/JAHA.120.018735.

28. Villuendas, R.; Martínez-Morillo, M.; Juncà, G.; Teniente-Serra, A.; Diez, C.; Heredia, S.; Riveros-Frutos, A.; Bayés-Genís, A.; Olivé, A. Usefulness of Cardiac Screening in Patients with Systemic Lupus Erythematosus and Anti-Ro/SSA Antibodies. *Lupus* **2021**, *30*, 1596–1602, doi:10.1177/09612033211027928.

29. Mostafavi, A.A.; Taassoarian, B.; Khadir, V.; Abbaszadeh, S.; Sanatkar, S.A.; Rafiei, M. Assessment of the Relationship Between Dose and Number of Effective Used Drugs on on QT Interval in Patients with Lupus. *Shiraz E Med J* **2020**, *21*, doi:10.5812/semj.83710.

30. Lazzerini, P.E.; Yue, Y.; Srivastava, U.; Fabris, F.; Capecchi, P.L.; Bertolozzi, I.; Bacarelli, M.R.; Morozzi, G.; Acampa, M.; Natale, M.; et al. Arrhythmogenicity of Anti-Ro/SSA Antibodies in Patients With Torsades de Pointes. *Circ Arrhythm Electrophysiol* **2016**, *9*, doi:10.1161/CIRCEP.115.003419.

31. Perez-García, L.F.; Estevez-García, I.O.; Moreno-Ramírez, M.; Félix, J.L.; Marquez-Velasco, R.; Iturralde, P.; Silveira, L.H.; Amezcua-Guerra, L.M. Anti-Ro52/TRIM21 Antibodies Are Associated with QT Interval Prolongation in Patients with Systemic Lupus Erythematosus. *ACR/ARHP Annual Meeting* **2016**.

32. Tufan, A.N.; Sag, S.; Oksuz, M.F.; Ermurat, S.; Coskun, B.N.; Gullulu, M.; Budak, F.; Baran, I.; Pehlivan, Y.; Dalkilic, E. Prolonged Tpeak–Tend Interval in Anti-Ro52 Antibody-Positive Connective Tissue Diseases. *Rheumatol Int* **2017**, *37*, 67–73, doi:10.1007/s00296-016-3488-1.

33. Pisoni, C.N.; Reina, S.; Arakaki, D.; Eimon, A.; Carrizo, C.; Borda, E. Elevated IL-1β Levels in Anti-Ro/SSA Connective Tissue Diseases Patients with Prolonged Corrected QTc Interval. *Clin Exp Rheumatol* **2015**, *33*, 715–720.

34. Sham, S.; Madheshwaran, M.; Tamilselvam, T.N.; Rajeswari, S. Correlation of QT Interval with Disease Activity in Newly Detected SLE Patients at Baseline and during Flare. *Indian J Rheumatol* **2015**, *10*, 121–124, doi:10.1016/j.injr.2015.03.010.

35. Massie, C.; Hudson, M.; Tatibouet, S.; Steele, R.; Huynh, T.; Fritzler, M.J.; Baron, M.; Pineau, C.A. Absence of an Association between Anti-Ro Antibodies and Prolonged QTc Interval in Systemic Sclerosis: A Multicenter Study of 689 Patients. *Semin Arthritis Rheum* **2014**, *44*, 338–344, doi:10.1016/j.semarthrit.2014.07.001.

36. Nomura, A.; Kishimoto, M.; Takahashi, O.; Deshpande, G.A.; Yamaguchi, K.; Okada, M. Prolongation of Heart Rate-Corrected QT Interval Is a Predictor of Cardiac Autonomic Dysfunction in Patients with Systemic Lupus Erythematosus. *Rheumatol Int* **2014**, *34*, 643–647, doi:10.1007/s00296-013-2718-z.

37. Bourré-Tessier, J.; Clarke, A.E.; Huynh, T.; Bernatsky, S.; Joseph, L.; Belisle, P.; Pineau, C.A. Prolonged Corrected QT Interval in Anti-Ro/SSA-Positive Adults with Systemic Lupus Erythematosus. *Arthritis Care Res (Hoboken)* **2011**, *63*, 1031–1037, doi:10.1002/acr.20470.

38. Lazzerini, P.E.; Capecchi, P.L.; Acampa, M.; Morozzi, G.; Bellisai, F.; Bacarelli, M.R.; Dragoni, S.; Fineschi, I.; Simpatico, A.; Galeazzi, M.; et al. Anti-Ro/SSA-Associated Corrected QT Interval Prolongation in Adults: The Role of Antibody Level and Specificity. *Arthritis Care Res (Hoboken)* **2011**, *63*, 1463–1470, doi:10.1002/acr.20540.

39. Lazzerini, P.E.; Capecchi, P.L.; Guideri, F.; Bellisai, F.; Selvi, E.; Acampa, M.; Costa, A.; Maggio, R.; Garcia-Gonzalez, E.; Bisogno, S.; et al. Comparison of Frequency of Complex Ventricular Arrhythmias in Patients with Positive versus Negative Anti-Ro/SSA and Connective Tissue Disease. *Am J Cardiol* **2007**, *100*, 1029–1034, doi:10.1016/j.amjcard.2007.04.048.

40. Lazzerini, P.E.; Acampa, M.; Guideri, F.; Capecchi, P.L.; Campanella, V.; Morozzi, G.; Galeazzi, M.; Marcolongo, R.; Laghi-Pasini, F. Prolongation of the Corrected QT Interval in Adult Patients with Anti-Ro/SSA–Positive Connective Tissue Diseases. *Arthritis Rheum* **2004**, *50*, 1248–1252, doi:10.1002/art.20130.

41. Behan, W.M.; Behan, P.O.; Gairns, J. Cardiac Damage in Polymyositis Associated with Antibodies to Tissue Ribonucleoproteins. *Heart* **1987**, *57*, 176–180, doi:10.1136/hrt.57.2.176.
